# Blunted heart rate response during dipyridamole stress SPECT is associated with cardiovascular outcomes in an all-comers cohort

**DOI:** 10.1186/s12872-026-06153-5

**Published:** 2026-06-23

**Authors:** Alexander Izhaki, Aviram Akuka, Alexey Migranov, Diklah Geva, Dina Vorobeichik, Ronen Rubinshtein

**Affiliations:** 1 Heart Institute, Edith Wolfson Medical Center, 62 Halochamim Street, Holon, 5810001 Israel; 2 Gray School of Medicine, Tel Aviv University, Tel-Aviv, Israel; 3 IntegriStat LTD, Tel-Aviv, Israel

**Keywords:** SPECT, Dipyridamole, Ischemic heart disease, Blunted heart rate response

## Abstract

**Background:**

Dipyridamole stress cardiac 99mTc-SPECT (DS-SPECT) is commonly used to detect myocardial ischemia. Blunted heart rate response (BHRR) during pharmacologic stress has been associated with adverse outcomes. This study evaluated the significance of BHRR in an unselected real-world DS-SPECT cohort and compared it with routine imaging findings.

**Methods:**

In this single-center retrospective cohort study, all adult patients who underwent dipyridamole stress SPECT without adjunct exercise between 2014 and 2017 were included. Clinical data were obtained from the electronic medical record and structured pre-test interviews, and patients were followed through September 2020.

**Results:**

Study population consisted of 388 patients, 227 (58.5%) had normal imaging results, and 245 (63.1%) exhibited BHRR. During a mean follow-up of four years, multivariable analysis found association of BHRR with cardiovascular death (HR 8.09, 95% CI:1.06–61.91, *p* < 0.05). Patients with abnormal DS-SPECT imaging and BHRR had a nearly fourfold increased risk of adverse cardiovascular events (HR 3.79, 95% CI: 1.24–11.63, *p* < 0.05). Moreover, individuals demonstrating both abnormal imaging results and BHRR experienced the highest rate of all-cause mortality (HR 2.93, 95% CI: 1.1–7.7, *p* < 0.01). Overall, BHRR significantly correlated with increased all-cause and cardiovascular mortality.

**Conclusions:**

In unselected patients referred to DS-SPECT, BHRR was associated with cardiovascular mortality. Moreover, in our population group, when combined with abnormal imaging results, BHRR significantly increases the association with DS-SPECT for cardiovascular morbidity and all-cause mortality. These findings may support future inclusion of BHRR in DS-SPECT studies.

**Graphical Abstract:**

**Figure:**
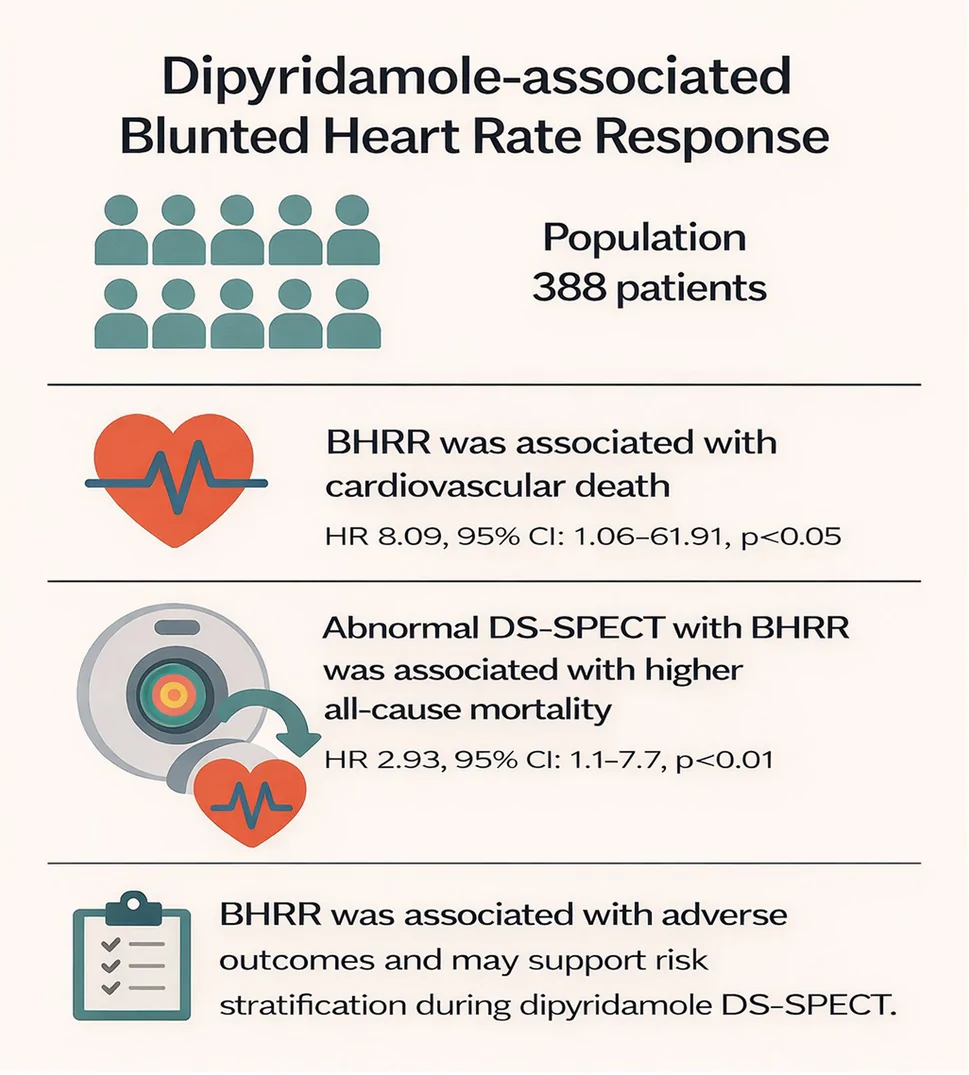

**Supplementary Information:**

The online version contains supplementary material available at https://doi.org/10.1186/s12872-026-06153-5.

## Introduction

99mTc Single Photon Emission Computed Tomography (SPECT) is widely used to assess myocardial ischemia in patients with coronary artery disease (CAD) [1–6]. Pharmacologic stress testing is typically reserved for those unable to exercise, or with left bundle-branch block or paced rhythms [1–3], as SPECT retains high sensitivity and specificity [7–11], with demonstrated cost-effectiveness in CAD detection [12–15]. One of the most used agents for pharmacologic stress SPECT is Dipyridamole, an indirect coronary vasodilator [16, 17], known to improve diagnostic yield [18, 19] while maintaining a strong safety profile [20].

Although low risk and normal SPECT test results are associated with low probability of major adverse cardiac events (MACE) [7, 8, 21, 22], in high risk populations (especially patients with: diabetes, atrial fibrillation, chronic renal impairment and elderly), a normal test result does not necessarily translate into low mortality and MACE risk [23–36]. Moreover, as the incidence of abnormal SPECT tests has progressively decreased, the proportion of these higher risk profile subjects among the testing population continues to grow [37–39], thus potentially negatively influencing the predictive value of the test.

Nevertheless, pharmacologic stress SPECT yields not only imaging findings but also important non-imaging prognostic information. A growing body of evidence indicates that a blunted heart rate response to vasodilator stress is an independent predictor of all-cause and cardiovascular mortality, providing incremental prognostic value beyond clinical and imaging variables [40–46], even in the setting of normal perfusion studies [47, 48]. While the proposed mechanisms underlying blunted heart rate response (BHRR) include cardiac autonomic neuropathy (CAN) [43, 49–52] and left ventricular dysfuntion [41].

As noted above, the proportion of normal SPECT studies has been steadily increasing [37–39]. At the same time, the population referred for testing has become increasingly comorbidity-rich, including a growing proportion of patients with atrial fibrillation (AF)/atrial flutter (AFL) and Cardiac Implantable Electronic Devices (CIED) 53, 54, 55, 56. This is clinically important because these patients are now commonly encountered in routine pharmacologic SPECT practice, yet they were often excluded from prior studies evaluating blunted heart rate response [10, 41, 44–47]. Their exclusion limits the generalizability of the existing literature, whereas their inclusion in the present study helps address an important real-world clinical gap regarding the prognostic value of BHRR in contemporary referral populations.

## Methods

### Objectives

To perform an “all-comers” study characterized by a diverse and contemporary comorbidity profile, reflecting the current patient population undergoing Dipyridamole induced Stress Cardiac SPECT (DS-SPECT). The primary objective is to evaluate the independent prognostic significance of BHRR and its added value when combined with imaging findings, in predicting cardiovascular events and mortality.

### Study design and population

This study is a single-center retrospective cohort study. All patients over 18 subjected to Dipyridamole induced cardiac SPECT stress test with no additional exercise, for any indication, from 2014 to 2017 in our center were included, and followed up until September 2020. All patients who underwent exercise stress DS-SPECT or combined exercise and pharmacological stress protocols were excluded. The patients were referred to for testing for any clinical indication, including suspected or confirmed coronary heart disease. All the tests were conducted in the same SPECT camera.

Patients’ data was acquired from their electronic medical record, coded according to the Ninth Revision of the International Classification of Diseases (ICD-9) and from pretest structured interviews. For all patients, age and gender, including the diagnosis of further chronic comorbidities: CAD, Diabetes Mellitus (DM), Hypertension (HTN) and End Stage Renal Failure (ESRF, glomerular filtration rate of less than 15 mL/min). AF/AFL and CIED status were identified from the electronic medical record and pre-test clinical data. In this retrospective dataset, detailed rhythm during stress testing, percentage of ventricular pacing, pacemaker dependence, rate-response activation, and device programming were not systematically available.

This study was approved by the local institutional review board with waiver of informed consent.

### Imaging test conduction and analysis

Imaging was performed using an Infinia Hawkeye 4 camera (GE healthcare), and processing was implemented by GE Xeleris workstation, with eight frames per cardiac cycle and iterative reconstruction with and without CT attenuation correction.

A same-day dipyridamole (stress) -rest ^99m^Tc sestamibi 10/30 mCi protocol was implemented [16]. In case stress images were interpreted as abnormal, additional 30 millicuries ^99m^Tc sestamibi were reinjected two hours after stress dose injection. Both post-stress and rest images were acquired one-hour post tracer injections.

All patients were under caffeine/xantines restriction regiment before the test and were ordered to stop the use of rate limiting agents, especially beta-blockers, 24 h before the test.

### Outcomes and variables

All subjects were assigned into four pre-defined DS-SPECT categories: Normal, Reversible (ischemic defects) only, Irreversible (fixed) defects only and Mixed type (both reversible and irreversible defects). Normal test results were defined as no ^99m^Tc uptake defects and post stress LVEF ≥ 50%. Heart-rate response (HRR) was calculated as [(peak heart rate − baseline heart rate) / baseline heart rate] × 100, and blunted heart-rate response (BHRR) was defined as HRR < 20%.

Our definition of BHRR with the 20% cutoff was based on previous similar studies [41, 44–46].

All patients were followed for the occurrence of two end-point outcome events: all-cause (AC) mortality and cardiovascular (CV) mortality. Events during 60 days after index DS-SPECT date were excluded to avoid test-driven events. It is worth mentioning that patients demonstrating reversible defects were admitted for early revascularization assessment.

Mortality was corroborated through the Ministry of Interior records. Information obtained from the patients’ medical electronic files included: the diagnosis and dates of CV events (CVE) - cardiovascular death, non-fatal myocardial infarction, stroke, coronary intervention and CIED implantation.

### Statistical analysis

Baseline characteristics were described by means and standard deviations (SD) for continuous variables and percentages for categorical variables. The comparison between different variables was performed using Student’s t-test for continuous variables, and Pearson chi-square for categorical variables. In addition, each variable’s effect on each study outcome was evaluated in a univariate Cox Proportional Hazard model and was tabulated and presented in a forest plot. Multivariable Cox Proportional Hazard regression was run to evaluate the adjusted effect of the variable of interest. Receiver-operating characteristic (ROC) analysis was also conducted to evaluate the best HRR cut-off as a predictor of all-cause mortality. The study significance level was set to 5% and no correction for multiple testing was conducted.

### Subgroup analysis

Subgroup analyses were conducted to evaluate the different characteristics and outcome of the cohort population according to heart rate response (BHRR vs. normal response). Because HRR may be less physiologically interpretable in patients with AF/AFL, paced rhythm, or rate-responsive devices, we performed a sensitivity analysis after excluding patients with AF/AFL or CIED. Event rates were compared between patients with BHRR and normal HRR. Similar subgroup analysis was conducted on the cohort dividing the patients by DS-SPECT results.

Finally, given the potential small number of cardiovascular death events, we performed a reduced-adjustment sensitivity Cox model including only age and sex to mitigate overfitting risk.

### Declaration of generative AI and AI-assisted technologies in the writing process

During the preparation of this work the author used CHATGPT in order to improve grammar. After using this tool, the author reviewed and edited the content as needed and takes full responsibility for the content of the published article.

## Results

### Study population

Study population comprised of 388 subjects with mean follow-up period lasted 1560 days (4.3 ± 1.5 years). Male comprised 212 (54.6%) of cohort, with 38 (9.8%) of patients after CIED implantation (four with ICD) and 27 (7.0%) with permanent atrial fibrillation/flutter. 227 (58.5%) of total patient population had normal DS-SPECT results, 29 (7.4%) had only reversible defects in the test, 83(21.6%) showed irreversible defects and 47 (12.1%) showed mixed results. Two patients could not be assigned to any category. 363 of the patients had LVEF information, post-stress LVEF ≤ 40% was observed in 28 (22.8%) of the 123 patients with numerically determined LVEF values, as post stress LVEF ≥ 50% represents 281(77.4% of the patients with known LVEF) patients. Abnormal Transient Ischemic dilatation (TID) was rare (9/388, 2.3%) and occurred exclusively in patients with abnormal perfusion imaging, no cases of abnormal TID were observed among normal scans (0/227).

The mean heart rate response was 16.1 with standard deviation of ±14.8. Blunted heart rate response was observed in 245 out of 388 patients with available data (63.1%), constituted a majority in every scan category, 140(61.6%), 15(52%), 56 (67.5%), and 31(66%) of the normal, reversible, fixed and mixed DS-SPECT, respectively.

The incidence of any cardiovascular event was 21.9% (85 patients). 90 patients (23.2%) have died during follow up period, as cardiovascular cause of death was confirmed in 20 patients (5.3%). The full population’s characteristics and outcomes are detailed in Table 1.

**Table 1:** Characteristics and Outcomes of study population

Variable	Cohort (*n* = 388)
Age (years)	73.4±9.6
Males (%)	212 (54.6)
Coronary artery disease (%)	200 (51.5)
CIED (%)	38 (9.8)
Atrial fibrillation/flutter (%)	27 (7.0)
Insulin dependent diabetes (%)	29 (7.6)
Hypertension (%)	294 (77.6)
End Stage Renal Disease (%)	12 (3.1)
Pre- test angina (%)	112 (28.9)
Basal heart rate (bpm)	69.6 ± 11.8
Peak heart rate (bpm)	80.4 ± 14.4
Heart rate response	16.1 ± 14.8
BHRR (%)	245 (63.1)
Normal DSPECT (%)	227 (58.5)
Reversible defects in DSPECT (%)	29 (7.4)
Fixed only DSPECT (%)	83 (21.6)
Mixed DSPECT (%)	47 (12.1)
Post stress LVEF ≥ 50% (% *n* = 363)	281 (77.4)
Transient Ischemic Dilation	9 (2.3%)
Cardiovascular event (%)	85 (21.9)
All cause death (%)	90 (23.2)
Cardiovascular death (%)	20 (5.3)

### Cox proportional-hazards regression analysis

Univariate analysis identified atrial fibrillation/flutter, insulin dependent diabetes and elevated basal HR as predictors of CV death. In this univariant analysis, BHRR was linked to CV death (HR 11.18, 95% CI: 1.5–83.54, *p* = 0.019) and AC death (HR 2.41, 95% CI: 1.43–4.03, *p* = 0.001). Abnormal DS-SPECT (fixed) was associated with CV death (HR 3.2, 95% CI: 1.32–7.72, *p* = 0.01) and AC death (HR 1.85, 95% CI: 1.19–2.88, *p* = 0.006). The heart rate response association with CV and AC death was statistically significant but the difference not clinically meaningful (HR 0.97, 95% CI: 0.94–0.99, *p* = 0.003 and HR 0.98, 95% CI: 0.96–0.99, *p* < 0.001 respectably). Moreover, in our analysis, no significant association was found between TID and any of the defined outcomes. Interestingly, the association between CV death and previously known cardiovascular disease was not statistically significant (Table 2).

**Table 2:** Predictors of all Cause and Cardiovascular death, univariate analysis

Death	Cardiovascular Death		All-cause Mortality	
Predictor	Confidence Interval	*p* value	Confidence Interval	*p* value
Age (year)	1.02 (0.97,1.07)	0.415	1.04 (1.01, 1.06)	0.01
Known coronary artery disease	1.32 (0.55,3.2)	0.535	1.81 (1.18, 2.78)	0.007
Atrial fib/flutter	4.12 (1.35,12.52)	0.013	1.92 (0.99,3.71)	0.052
Insulin dependent diabetes	6.35 (2.13, 18.92)	0.001	3.12 (1.74, 5.58)	< 0.001
Basal heart rate	1.03 (1.0,1.07)	0.053	1.0 (0.98,1.02)	0.869
Heart rate response (bpm)	0.97(0.94,0.99)	0.003	0.98(0.96,0.99)	< 0.001
Peak heart rate	0.99 (0.96,1.02)	0.454	0.98 (0.96,0.99)	0.004
BHRR	11.18 (1.5,83.54)	0.019	2.41(1.43,4.03)	0.001
Post stress LVEF ≥ 50%	0.41 (0.16,1.04)	0.06	0.53 (0.38,0.85)	0.008
Transient Ischemic Dilation	2.03 (0.27–15.14)	0.491	0.92 (0.23–3.74)	0.908
Normal DSPECT	0.34 (0.13.0.85)	0.021	0.62 (0.41,0.93)	0.022
Fixed DSPECT	3.2 (1.32,7.72)	0.01	1.85 (1.19,2.88)	0.006
Reversible DSPECT	0	0.997	0.6 (0.22,1.65)	0.324

In multivariable analysis, BHRR has shown independent association with CV death (HR 8.09, CI: 1.06–61.91, *p* < 0.05) but not with AC death (HR 1.11, 95% CI: 0.57–2.16) or first CV event (HR 0.92, 95% CI: 0.44–1.92). The combined exposure of BHRR and abnormal DS-SPECT was associated with first cardiovascular events (HR 3.79, 95% CI: 1.24–11.63, *p* < 0.05), but not with all-cause death (HR 2.78, 95% CI: 0.89–8.68). This combined-exposure variable should not be interpreted as a formal interaction term, as formal interaction testing for cardiovascular death was not feasible because of sparse events (Table 3).

**Table 3:** Multivariate Cohort Outcomes Analysis

	CV death	AC death	1st CVE at > 60 days
BHRR	8.09* (1.06,61.91)	1.11 (0.57,2.16)	0.92 (0.44,1.92)
DSPECT – abnormal vs. normal	2.75(0.94,8.07)	0.77(0.27,2.20)	0.81 (0.30,2.20)
Age	1.03 (0.98,1.08)	1.06*** (1.03,1.08)	1.00 (0.97,1.02)
male vs. female	0.95 (0.35, 2.58)	1.05 (0.67, 1.64)	1.10 (0.68, 1.78)
Diabetes: no vs. insulin therapy	3.99* (1.23,12.91)	2.72** (1.43,5.16)	4.64*** (2.36,9.11)
End stage renal disease	2.71 (0.29,25.06)	6.27*** (2.63, 14.95)	2.30 (0.79,6.70)
Transient Ischemic Dilation	1.02 (0.13–7.88)	0.65 (0.16–2.68)	1.98 (0.78–5.00)
BHRR and abnormal DSPECT°		2.78 (0.89, 8.68)	3.79∗ (1.24, 11.63)

*p < 0.05

**p < 0.01

***p < 0.001

° This row represents the combined exposure of BHRR with abnormal DS-SPECT: CV death outcome interaction was impossible to calculate due to lack of events in each relevant cell

Further multivariant analysis reveals that BHRR was associated with both all cause death (HR 2.93, CI: 1.1–7.7, *p* < 0.05) and CVE (HR 3.43, CI: 1.4–8.3, *p* < 0.005) among the abnormal DS-SPECT patients, but not in patients with normal imaging results (Table 4).

**Table 4:** Multivariable analysis by DSPECT status

Covariate	Normal DSPECT						Abnormal DSPECT					
	Dependent variables: Hazard Ratios with 95% Confidence Intervals											
	All-cause death			1st CVE			All-cause death			1st CVE		
	HR	CI		HR	CI		HR	CI		HR	CI	
BHRR	1.16	0.6	2.2	0.87	0.4	1.90	2.93*	1.1	7.7	3.43**	1.4	8.3
Age	1.04*	1.0	1.1	0.99	0.96	1.03	1.07***	1.0	1.1	1.0	0.97	1.04
Diabetes treated with insulin°	2.8*	1.4	5.2	8.5***	2.9	25.1	2.4*	1.1	5.3	3.06**	1.3	7.06
End stage renal disease	4.5**	2.6	15	2.3	0.62	8.4	11.6**	2.4	55.6	1.63	0.2	13.1

∗*p* < 0.05

∗∗*p* < 0.01

∗∗∗*p* < 0.001

°Comparing orally treated diabetics vs. non diabetics

**Fig. 1:**
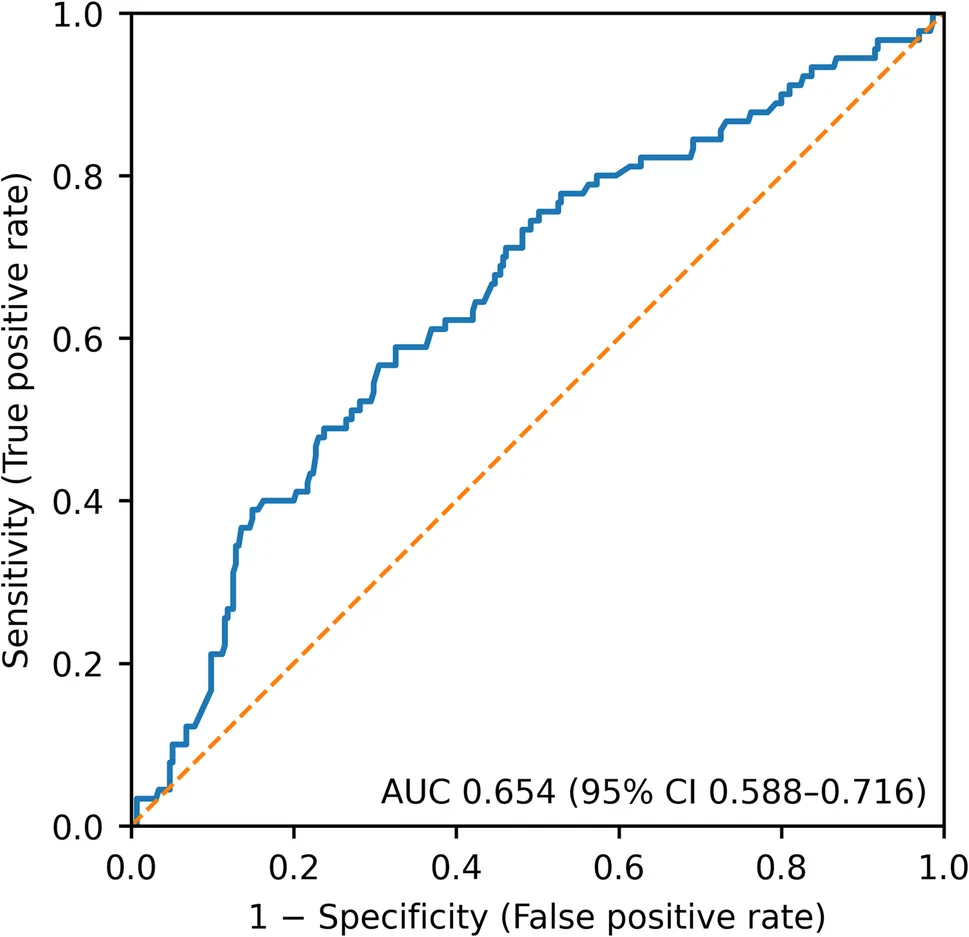
ROC curve of heart rate response (HRR) for prediction of all-cause mortality. Receiver-operating characteristic (ROC) analysis was performed using the percent increase in heart rate during dipyridamole stress. Lower HRR values indicate higher risk. The area under the ROC curve (AUC) was 0.654 (95% CI 0.588–0.716). The optimal HRR cutoff was 16.5% with sensitivity of 75.6% and specificity of 49.7%, compare with the 20% cutoff yielded sensitivity 81.1% and specificity 37.6%

In ROC analysis evaluating HRR as a predictor of all-cause mortality, discriminatory performance was modest (AUC 0.654, 95% CI 0.588–0.716). The optimal HRR cutoff was 16.5% increase, providing a sensitivity of 75.6% and specificity of 49.7%, comparing the common HRR cutoff of 20%, with increased sensitivity to 81.1% but reduced specificity to 37.6% (Fig. 1).

### Subgroup analysis

Our subgroup analyses, as detailed in the supplementary material, revealed similar results to the main dataset, indicating internal consistency of the results. AC mortality, CV death and CVE, were more frequent among BHRR patients. In the sensitivity cohort analysis excluding patients with AF/AFL or CIED, 199 patients had BHRR and 130 had normal HRR. All-cause death occurred in 52/199 patients with BHRR versus 16/130 with normal HRR, cardiovascular death in 13/199 versus 1/130, and first cardiovascular event in 50/199 versus 22/130. These findings were directionally consistent with the main cohort; however, they should be interpreted as sensitivity findings rather than proof of robustness (Table S1-2).

Another subgroup analysis, assessing the different clinical descriptors by DS-SPECT categories, found that normal results group, compared to fixed and mixed groups, demonstrated lower CV death (3% vs.11.4% vs. 8.5%, *p* = 0.04) and all-cause mortality (19.8% vs. 34.1% vs. 27.7%, *p* = 0.03). In abnormal DS-SPECT patients, BHRR was associated with AC death (HR 2.93, 95% CI: 1.1–7.7, *p* < 0.05) and CV events (HR 3.43, 95% CI: 1.4–8.3, *p* < 0.005). No such associations were observed in normal DS-SPECT patients (Table S3). The low rates in the reversible group are due to low sample size (*n* = 29).

Given the limited number of cardiovascular deaths, the fully adjusted cardiovascular-death model should be interpreted as exploratory. In a reduced Cox model adjusted only for age and sex, BHRR remained associated with cardiovascular death (HR 9.52, 95% CI 1.27–71.33, *p* = 0.028), although the confidence interval remained wide (Table S4).

## Discussion

This is a unique cohort retrospective study aimed at evaluating association between the BHRR and DS-SPECT imaging results and cardiovascular mortality and morbidity in an all-comers population with significant comorbidities. The study population is characterised by high rates of octogenarians, diabetic patient, patients with atrial fibrillation/flutter and CIED implanted subjects which were often excluded from previous studies [10, 41–47]. Our cohort BHRR rate (64%), was grossly consistent with previous studies [44–47], while the relatively high incidence can be explained by the higher rates of diabetes and older age in our study population. As previously described in the literature, older age and diabetes are known to be associated with Cardiac Autonomous Neuropathy (CAN), which is characterized by chronotropic incompetence (may manifest as BHRR) and a reduced long-term survival rates [49–52].

CV mortality among patients with normal DS-SPECT imaging results in our study was low (averaging less than 1% per one year follow-up), in accordance with the known literature [7–11]. The relatively higher rate of AC mortality (19.8% at end of follow up period) among the normal DS-SPECT patients can be attributed to the high rates of comorbidities in our study population, whereas sub-optimal (attenuated) “stress” heart rates may also have partially contributed to false–negative results [57].

In our study, BHRR appears to be independently associated with cardiovascular death in multivariate analysis, this observed associations with BHRR align with prior studies showing its link to mortality in vasodilator stress testing [40–47], though some reported effects even in normal perfusion, which was not replicated here (possibly due to power issues). Our findings were further cemented by our subgroup analysis that demonstrated significate increase in CV and AC mortality in patients with BHRR, repeated in further analysis of patients without comorbidities of AF/AFL and CIED. Importantly, however, the association between BHRR and cardiovascular death should be interpreted cautiously because only 20 cardiovascular deaths occurred and the adjusted estimate was accompanied by wide confidence intervals. Although the direction of association was consistent across model specifications, including the reduced age- and sex-adjusted Cox model, this finding is best viewed as exploratory rather than definitive and requires confirmation in larger cohorts. Likewise, the lack of a significant association between BHRR and outcomes in the normal DS-SPECT subgroup should not be interpreted solely as confirmation of the high negative predictive value of a normal study. Event rates in this subgroup were low, including only 7 cardiovascular deaths among 227 patients, and limited statistical power may therefore have contributed to a type II error.

Additionally, the association of BHRR with adverse outcome events includes reduced post stress LVEF, lower survival rate and a fourfold increase in CVE among patients with abnormal DS-SPECT, implying the possible added value of BHRR over DS-SPECT imaging test results alone [58, 59]. However, because quantitative perfusion indices were not available, we cannot exclude the possibility that the association of BHRR with outcomes was partly related to ischemic severity not fully captured by the qualitative DS-SPECT categories.

Moreover, in contrast to a previous study [48], impact of BHRR on AC and CV death rates among our normal DS-SPECT patients did not reach statistical significance, thus retaining the well-known high negative predictive value of a normal DS-SPECT result. Based on comparable rates of non-fatal MI and revascularizations, in either BHRR presence or absence subsets, Mathur et al. [44] suggested that that BHRR is specifically associated with cardiac death by non-ischemic mechanisms like ventricular arrhythmias, this may explain our results’ discrepancy of significant higher CV death rates in patients with BHRR compared with patients with normal HRR, contrary to almost no difference in other CVE.

### Strengths and limitations

The major strength of the study is by including contemporary, higher risk, all-comer population, moreover, it includes results and data on patients that were previously not included in similar studies. The study also has its strength in its relatively long follow-up period and the assessment of both imaging and BHRR results.

The study’s main limitation is its retrospective nature and its single center design, which may have resulted in selection bias, as the reliance on ICD 9 codes and interviews may also result in incomplete data. Second, our study population is relatively small, although population size was satisfactory in most cases for reaching statistical significance, with only 388 patients and rare CV deaths (20 events), the analyses can be underpowered, leading to wide confidence intervals and inability to compute some interactions. Moreover, DS-SPECT categories were qualitative without quantitative metrics and LVEF data was missing in 25 patients, which reflects another limitation regarding the imaging results. The lack of quantitative perfusion/ischemic burden metrics may limit our ability to assess whether BHRR provides prognostic information beyond the magnitude of ischemia itself. An additional limitation relates to rhythm and device status, although patients with AF/AFL and CIEDs were included to reflect real-world DS-SPECT practice, HRR may not have the same physiological meaning in patients with irregular rhythm, paced rhythm, pacemaker dependence, or rate-responsive device programming. This information was not systematically available in this retrospective dataset. Therefore, the AF/AFL/CIED-excluded analysis was presented as a sensitivity analysis and should not be interpreted as definitive proof of robustness. Finally, although patients were instructed to withhold rate limiting drugs and caffeine, we lacked the full data on baseline medication exposure and caffeine blood levels, so residual caffeine exposure or medication confounding cannot be excluded.

## Conclusions

In conclusion, we conducted an “all-comers” cohort study reflecting the contemporary population referred for DS-SPECT, characterized by older age and a high prevalence of diabetes, AF, and CIED. In this cohort, the presence of BHRR was associated with cardiovascular mortality and, among patients with abnormal imaging, with all-cause mortality and cardiovascular events. These findings suggest that DS-SPECT results should not be interpreted in isolation and that incorporation of BHRR may improve risk stratification. However, because quantitative SPECT metrics were unavailable, whether BHRR adds prognostic information beyond ischemic burden remains uncertain and larger prospective cohort studies are needed to confirm these findings.

## Supplementary Information

Supplementary Material 1.

## Data Availability

The data that support the findings of this study are not publicly available due to privacy considerations, but are available from the corresponding author upon reasonable request.

## Abbreviations

99mTc: Technetium–99 m
AC: All–Cause (e.g., all–cause mortality)
AF: Atrial Fibrillation
AFL: Atrial Flutter
BHRR: Blunted Heart Rate Response
CAD: coronary artery disease
CAN: Cardiac Autonomic Neuropathy
CIED: Cardiac Implantable Electronic Device
CV: Cardiovascular
CVE: Cardiovascular Events
DM: Diabetes Mellitus
DS: SPECT–Dipyridamole Stress Single Photon Emission Computed Tomography
ESRF: End Stage Renal Failure
HR: Heart Rate
HRR: Heart Rate Response
HTN: Hypertension
ICD: Implantable Cardioverter–Defibrillator
ICD: 9–International Classification of Diseases, 9th Revision
LVEF: Left Ventricular Ejection Fraction
MACE: Major Adverse Cardiac Events
MI: Myocardial Infarction
SD: Standard Deviation

## References

[1] Henzlova MJ, Cerqueira MD, Mahmarian JJ, Yao SS. Quality Assurance Committee of the American Society of Nuclear Cardiology. Stress protocols and tracers. J Nucl Cardiol. 2006;13(6):e80–90. 17174798 10.1016/j.nuclcard.2006.08.011

[2] Druz RS. Current advances in vasodilator pharmacological stress perfusion imaging. Semin Nucl Med. 2009;39(3):204–9. 19341840 10.1053/j.semnuclmed.2008.12.003

[3] Hendel RC, Berman DS, Di Carli MF, et al. ACCF/ASNC/ACR/AHA/ASE/SCCT/SCMR/SNM 2009 appropriate use criteria for cardiac radionuclide imaging: a report of the American College of Cardiology Foundation Appropriate Use Criteria Task Force, the American Society of Nuclear Cardiology, the American College of Radiology, the American Heart Association, the American Society of Echocardiography, the Society of Cardiovascular Computed Tomography, the Society for Cardiovascular Magnetic Resonance, and the Society of Nuclear Medicine. Circulation. 2009;119(22):e561-e587. 10.1161/CIRCULATIONAHA.109.192519 19451357

[4] Knuuti J, Ballo H, Juarez-Orozco LE, et al. The performance of non-invasive tests to rule-in and rule-out significant coronary artery stenosis in patients with stable angina: a meta-analysis focused on post-test disease probability. Eur Heart J. 2018;39(35):3322–30. 29850808 10.1093/eurheartj/ehy267

[5] Gulati M, Levy PD, Mukherjee D, et al. 2021 AHA/ACC/ASE/CHEST/SAEM/SCCT/SCMR Guideline for the Evaluation and Diagnosis of Chest Pain: A Report of the American College of Cardiology/American Heart Association Joint Committee on Clinical Practice Guidelines. Circulation. 2021;144(22):e368–454. 34709879 10.1161/CIR.0000000000001029

[6] Vrints C, Andreotti F, Koskinas KC, et al. 2024 ESC Guidelines for the management of chronic coronary syndromes. Eur Heart J. 2024;45(36):3415–537. 39210710 10.1093/eurheartj/ehae177

[7] Underwood SR, Anagnostopoulos C, Cerqueira M, et al. Myocardial perfusion scintigraphy: the evidence. Eur J Nucl Med Mol Imaging. 2004;31(2):261–91. 15129710 10.1007/s00259-003-1344-5 PMC2562441

[8] Marcassa C, Bax JJ, Bengel F, et al. Clinical value, cost-effectiveness, and safety of myocardial perfusion scintigraphy: a position statement. Eur Heart J. 2008;29(4):557–63. 18202253 10.1093/eurheartj/ehm607

[9] Medical Advisory Secretariat. Single photon emission computed tomography for the diagnosis of coronary artery disease: an evidence-based analysis. Ont Health Technol Assess Ser. 2010;10(8):1–64. PMC3377554 23074411

[10] Cantoni V, Green R, Acampa W, et al. Diagnostic performance of myocardial perfusion imaging with conventional and CZT single-photon emission computed tomography in detecting coronary artery disease: A meta-analysis. J Nucl Cardiol. 2021;28(2):698–715. 31089962 10.1007/s12350-019-01747-3

[11] Driessen RS, van Diemen PA, Raijmakers PG, et al. Functional stress imaging to predict abnormal coronary fractional flow reserve: the PACIFIC 2 study. Eur Heart J. 2022;43(33):3118–28. 35708168 10.1093/eurheartj/ehac286 PMC9433308

[12] Underwood SR, Godman B, Salyani S, Ogle JR, Ell PJ. Economics of myocardial perfusion imaging in Europe–the EMPIRE Study. Eur Heart J. 1999;20(2):157–66. 10099913 10.1053/euhj.1998.1196

[13] Heller GV, Stowers SA, Hendel RC, et al. Clinical value of acute rest technetium-99m tetrofosmin tomographic myocardial perfusion imaging in patients with acute chest pain and nondiagnostic electrocardiograms. J Am Coll Cardiol. 1998;31(5):1011–7. 9562001 10.1016/s0735-1097(98)00057-6

[14] Shaw LJ, Hachamovitch R, Berman DS, et al. The economic consequences of available diagnostic and prognostic strategies for the evaluation of stable angina patients: an observational assessment of the value of precatheterization ischemia. Economics of Noninvasive Diagnosis (END) Multicenter Study Group. J Am Coll Cardiol. 1999;33(3):661–9. 10080466 10.1016/s0735-1097(98)00606-8

[15] Shaw LJ, Heller GV, Travin MI, et al. Cost analysis of diagnostic testing for coronary artery disease in women with stable chest pain. Economics of Noninvasive Diagnosis (END) Study Group. J Nucl Cardiol. 1999;6(6):559–69. 10608582 10.1016/s1071-3581(99)90091-0

[16] Henzlova MJ, Duvall WL, Einstein AJ, Travin MI, Verberne HJ. ASNC imaging guidelines for SPECT nuclear cardiology procedures: Stress, protocols, and tracers. J Nucl Cardiol. 2016;23(3):606–39. 26914678 10.1007/s12350-015-0387-x

[17] Hendel RC, Ramirredy A. Exercise and Pharmacological Stress Testing. Chapter 8. Nuclear Cardiology’ pages109-112. Practical Applications. 3rd Edition. McGraw Hill Education; 2018.

[18] Matzer L, Kiat H, Wang FP, Van Train K, Germano G, Friedman J, Berman DS. Pharmacologic stress dual-isotope myocardial perfusion single-photon emission computed tomography. Am Heart J. 1994;128(6 Pt 1):1067–76. 7985586 10.1016/0002-8703(94)90735-8

[19] Gholoobi A, Ayati N, Baghyari A, Mouhebati M, Atar B, Dabbagh Kakhki VR. Relationship between gated myocardial perfusion SPECT findings and hemodynamic, electrocardiographic, and heart rate changes after Dipyridamole infusion. Int J Cardiovasc Imaging. 2017;33(6):951–6. 28150082 10.1007/s10554-017-1074-6

[20] Lette J, Tatum JL, Fraser S, et al. Safety of dipyridamole testing in 73,806 patients: the Multicenter Dipyridamole Safety Study. J Nucl Cardiol. 1995;2(1):3–17. 9420757 10.1016/s1071-3581(05)80003-0

[21] skander S, Iskandrian AE. Risk assessment using single-photon emission computed tomographic technetium-99m sestamibi imaging. J Am Coll Cardiol. 1998;32(1):57–62. 9669249 10.1016/s0735-1097(98)00177-6

[22] Shaw LJ, Hage FG, Berman DS, Hachamovitch R, Iskandrian A. Prognosis in the era of comparative effectiveness research: where is nuclear cardiology now and where should it be? J Nucl Cardiol. 2012;19(5):1026–43. 22760523 10.1007/s12350-012-9593-y

[23] Hachamovitch R, Berman DS, Shaw LJ, et al. Incremental prognostic value of myocardial perfusion single photon emission computed tomography for the prediction of cardiac death: differential stratification for risk of cardiac death and myocardial infarction. Circulation. 1998;97(6):535–43. 9494023 10.1161/01.cir.97.6.535

[24] Giri S, Shaw LJ, Murthy DR, et al. Impact of diabetes on the risk stratification using stress single-photon emission computed tomography myocardial perfusion imaging in patients with symptoms suggestive of coronary artery disease. Circulation. 2002;105(1):32–40. 11772873 10.1161/hc5001.100528

[25] Abidov A, Hachamovitch R, Rozanski A, et al. Prognostic implications of atrial fibrillation in patients undergoing myocardial perfusion single-photon emission computed tomography. J Am Coll Cardiol. 2004;44(5):1062–70. 15337220 10.1016/j.jacc.2004.05.076

[26] Askew JW, Miller TD, Hodge DO, Gibbons RJ. The value of myocardial perfusion single-photon emission computed tomography in screening asymptomatic patients with atrial fibrillation for coronary artery disease. J Am Coll Cardiol. 2007;50(11):1080–5. 17825719 10.1016/j.jacc.2007.05.035

[27] Al-Mallah MH, Hachamovitch R, Dorbala S, Di Carli MF. Incremental prognostic value of myocardial perfusion imaging in patients referred to stress single-photon emission computed tomography with renal dysfunction. Circ Cardiovasc Imaging. 2009;2(6):429–36. 19920040 10.1161/CIRCIMAGING.108.831164

[28] Smit MD, Tio RA, Slart RH, Zijlstra F, Van Gelder IC. Myocardial perfusion imaging does not adequately assess the risk of coronary artery disease in patients with atrial fibrillation. Europace. 2010;12(5):643–8. 20022875 10.1093/europace/eup404

[29] Murthy VL, Naya M, Foster CR, et al. Improved cardiac risk assessment with noninvasive measures of coronary flow reserve. Circulation. 2011;124(20):2215–24. 22007073 10.1161/CIRCULATIONAHA.111.050427 PMC3495106

[30] Nucifora G, Schuijf JD, van Werkhoven JM, et al. Relationship between obstructive coronary artery disease and abnormal stress testing in patients with paroxysmal or persistent atrial fibrillation. Int J Cardiovasc Imaging. 2011;27(6):777–85. 20953841 10.1007/s10554-010-9725-x PMC3144360

[31] Nair SU, Ahlberg AW, Mathur S, Katten DM, Polk DM, Heller GV. The clinical value of single photon emission computed tomography myocardial perfusion imaging in cardiac risk stratification of very elderly patients (≥ 80 years) with suspected coronary artery disease. J Nucl Cardiol. 2012;19(2):244–55. 22071954 10.1007/s12350-011-9477-6

[32] Nudi F, Biondi-Zoccai G, Schillaci O, et al. Prognostic accuracy of myocardial perfusion imaging in octogenarians. J Nucl Cardiol. 2018;25(4):1342–9. 29094297 10.1007/s12350-017-1102-x

[33] Charytan DM, Skali H, Shah NR, et al. Coronary flow reserve is predictive of the risk of cardiovascular death regardless of chronic kidney disease stage. Kidney Int. 2018;93(2):501–9. 29032954 10.1016/j.kint.2017.07.025 PMC6381827

[34] Wang FP, Amanullah AM, Kiat H, Friedman JD, Berman DS. Diagnostic efficacy of stress technetium 99m-labeled sestamibi myocardial perfusion single-photon emission computed tomography in detection of coronary artery disease among patients over age 80. J Nucl Cardiol. 1995;2(5):380–8. 9420817 10.1016/s1071-3581(05)80025-x

[35] Gentile R, Vitarelli A, Schillaci O, et al. Diagnostic accuracy and prognostic implications of stress testing for coronary artery disease in the elderly. Ital Heart J. 2001;2(7):539–45. 11501963

[36] Lima RS, De Lorenzo A, Pantoja MR, Siqueira A. Incremental prognostic value of myocardial perfusion 99m-technetium-sestamibi SPECT in the elderly. Int J Cardiol. 2004;93(2–3):137–43. 14975539 10.1016/S0167-5273(03)00149-9

[37] Rozanski A, Gransar H, Hayes SW, et al. Temporal trends in the frequency of inducible myocardial ischemia during cardiac stress testing: 1991 to 2009. J Am Coll Cardiol. 2013;61(10):1054–65. 23473411 10.1016/j.jacc.2012.11.056

[38] Beller GA. How Should We Interpret the Decrease in Annual Volume of Stress Imaging Tests for Evaluation of Suspected or Known Coronary Artery Disease With Fewer High-Risk Test Results? Circ Cardiovasc Imaging. 2017;10(7):e006702. 28687540 10.1161/CIRCIMAGING.117.006702

[39] Al Badarin FJ, Chan PS, Spertus JA, et al. Temporal trends in test utilization and prevalence of ischaemia with positron emission tomography myocardial perfusion imaging. Eur Heart J Cardiovasc Imaging. 2020;21(3):318–25. 31292618 10.1093/ehjci/jez159 PMC7778342

[40] Lauer MS, Francis GS, Okin PM, Pashkow FJ, Snader CE, Marwick TH. Impaired chronotropic response to exercise stress testing as a predictor of mortality. JAMA. 1999;281(6):524–9. 10022108 10.1001/jama.281.6.524

[41] Lee KH, Yoon JK, Lee MG, Lee SH, Lee WR, Kim BT. Dipyridamole myocardial SPECT with low heart rate response indicates cardiac autonomic dysfunction in patients with diabetes. J Nucl Cardiol. 2001;8(2):129–35. 11295689 10.1067/mnc.2001.111798

[42] Abidov A, Hachamovitch R, Hayes SW, et al. Prognostic impact of hemodynamic response to adenosine in patients older than age 55 years undergoing vasodilator stress myocardial perfusion study. Circulation. 2003;107(23):2894–9. 12796141 10.1161/01.CIR.0000072770.27332.75

[43] Hage FG, Iskandrian AE. Heart rate response during vasodilator stress myocardial perfusion imaging: Mechanisms and implications. J Nucl Cardiol. 2010;17(4):536–9. 20506008 10.1007/s12350-010-9248-9

[44] Mathur S, Shah AR, Ahlberg AW, Katten DM, Heller GV. Blunted heart rate response as a predictor of cardiac death in patients undergoing vasodilator stress technetium-99m sestamibi gated SPECT myocardial perfusion imaging. J Nucl Cardiol. 2010;17(4):617–24. 20490960 10.1007/s12350-010-9242-2

[45] Gorur GD, Ciftci EA, Kozdag G, et al. Reduced heart rate response to dipyridamole in patients undergoing myocardial perfusion SPECT. Ann Nucl Med. 2012;26(8):609–15. 22714113 10.1007/s12149-012-0618-z

[46] Cortigiani L, Carpeggiani C, Landi P, Raciti M, Bovenzi F, Picano E. Usefulness of Blunted Heart Rate Reserve as an Imaging-Independent Prognostic Predictor During Dipyridamole Stress Echocardiography. Am J Cardiol. 2019;124(6):972–7. 31324358 10.1016/j.amjcard.2019.06.017

[47] Bhatheja R, Francis GS, Pothier CE, Lauer MS. Heart rate response during dipyridamole stress as a predictor of mortality in patients with normal myocardial perfusion and normal electrocardiograms. Am J Cardiol. 2005;95(10):1159–64. 15877986 10.1016/j.amjcard.2005.01.042

[48] Iqbal FM, Al Jaroudi W, Sanam K, et al. Reclassification of cardiovascular risk in patients with normal myocardial perfusion imaging using heart rate response to vasodilator stress. Am J Cardiol. 2013;111(2):190–5. 23111139 10.1016/j.amjcard.2012.09.013

[49] Doulgerakis D, Moyssakis I, Kapelios CJ, et al. Cardiac Autonomic Neuropathy Predicts All-Cause and Cardiovascular Mortality in Patients with End-Stage Renal Failure: A 5-Year Prospective Study. Kidney Int Rep. 2017;2(4):686–94. 29142986 10.1016/j.ekir.2017.03.002 PMC5678628

[50] Ewing DJ, Martyn CN, Young RJ, Clarke BF. The value of cardiovascular autonomic function tests: 10 years experience in diabetes. Diabetes Care. 1985;8(5):491–8. 4053936 10.2337/diacare.8.5.491

[51] Taskiran M, Fritz-Hansen T, Rasmussen V, Larsson HB, Hilsted J. Decreased myocardial perfusion reserve in diabetic autonomic neuropathy. Diabetes. 2002;51(11):3306–10. 12401723 10.2337/diabetes.51.11.3306

[52] de Souza R, Machado L, Azevedo AB, De Lorenzo A. Predictors of abnormal heart rate response to dipyridamole in patients undergoing myocardial perfusion SPECT. Ann Nucl Med. 2011;25(1):7–11. 10.1007/s12149-010-0420-8 20931306

[53] Valzania C, Torbica A, Tarricone R, Leyva F, Boriani G. Implant rates of cardiac implantable electrical devices in Europe: A systematic literature review. Health Policy. 2016;120(1):1–15. 26632502 10.1016/j.healthpol.2015.11.001

[54] Valzania C, Bonfiglioli R, Fallani F, et al. Single-photon cardiac imaging in patients with cardiac implantable electrical devices. J Nucl Cardiol. 2022;29(2):633–41. 33241474 10.1007/s12350-020-02436-2

[55] Krijthe BP, Kunst A, Benjamin EJ, et al. Projections on the number of individuals with atrial fibrillation in the European Union, from 2000 to 2060. Eur Heart J. 2013;34(35):2746–51. 23900699 10.1093/eurheartj/eht280 PMC3858024

[56] Benjamin EJ, Muntner P, Alonso A, American Heart Association Council on Epidemiology and Prevention Statistics Committee and Stroke Statistics Subcommittee, et al. Heart Disease and Stroke Statistics-2019 Update: A Report from the American Heart Association. Circulation. 2019;139(10):e56–528. 30700139 10.1161/CIR.0000000000000659

[57] Gimelli A, Liga R, Coceani M, Quaranta A, Emdin M, Marzullo P. Chronotropic response to vasodilator-stress in patients submitted to myocardial perfusion imaging: impact on the accuracy in detecting coronary stenosis. Eur J Nucl Med Mol Imaging. 2015;42(12):1903–11. 26194718 10.1007/s00259-015-3129-z

[58] Cortigiani L, Carpeggiani C, Landi P, Raciti M, Bovenzi F, Picano E. Prognostic Value of Heart Rate Reserve in Patients with Permanent Atrial Fibrillation during Dipyridamole Stress Echocardiography. Am J Cardiol. 2020;125(11):1661–5. 32273056 10.1016/j.amjcard.2020.02.046

[59] Sharir T, Germano G, Kavanagh PB, et al. Incremental prognostic value of post-stress left ventricular ejection fraction and volume by gated myocardial perfusion single photon emission computed tomography. Circulation. 1999;100(10):1035–42. 10477527 10.1161/01.cir.100.10.1035

