# Supplementary material for: Blunted heart rate response during dipyridamole stress SPECT is associated with cardiovascular outcomes in an all-comers cohort
Source: BMC Cardiovasc Disord. 2026 Jun 23;26:778. doi: 10.1186/s12872-026-06153-5 (PMC13551835; doi:10.1186/s12872-026-06153-5)
Supplement: Supplementary file 1 [file 12872_2026_6153_MOESM1_ESM.docx]

Table S1: Cohort population analysis by HRR

| Pts  Variable | Overall  n=388 (%) | BHRR  n=245 (%) | Normal HRR  n=143 (%) | P-value |
| --- | --- | --- | --- | --- |
| Demographics | | | | |
| Age (mean ±SD) | 73.4 ±9.6 | 74.1 ±9.8 | 72.2 ±9.2 | **0.05** |
| Females n (%) | 176 (45.4) | 104 (42.4) | 72 (50.3) | 0.16 |
| Coronary artery disease | 200 (51.5) | 128 (52.2) | 72 (50.3) | 0.8 |
| Diabetes treated with insulin | 29 (7.6) | 25 (10.3) | 4 (2.9) | **0.003** |
| End stage renal disease | 12 (3.1) | 11 (4.5) | 1 (0.7) | 0.08 |
| Atrial. fib/flutter. | 27 (7.0) | 22 (9.0) | 5 (3.5) | 0.07 |
| CIED | 38 (9.8) | 29 (11.8) | 9 (6.3) | 0.08 |
| DSPECT: n (%) | | | | |
| Normal | 227(59.0) | 140(57.9) | 87(60.8) | 0.52 |
| Stress only | 163 (42.0) | 104 (42.4) | 59 (41.3) | 0.9 |
| Fixed DSPECT | 83 (21.6) | 57 (23.3) | 26(18.9) | 0.19 |
| Fixed & Mixed | 129 (33.2) | 87(35.5) | 42(29.4) | 0.22 |
| Post stress normal | 281 (77.4) | 164 (72.9) | 117 (84.8) | **0.012** |
| Outcomes n (%) | | | | |
| All cause death | 90 (23.3) | 72 (29.4) | 18 (12.7) | **<0.001** |
| Cardiovascular death | 20 (5.3) | 19 (7.9) | 1 (0.7) | **0.005** |
| 1st Cardiovascular event | 85 (22.4) | 61 (25.5) | 24 (17.1) | 0.07 |

Table S2: Subgroup analysis for patients without CIED/AF by HRR

| Variable | BHRR  n=199 (%) | Normal HRR  n=130 (%) | p-value |
| --- | --- | --- | --- |
| Age, mean ± SD | 73.5±10.11 | 72.4 ±9.2 | 0.35 |
| Diabetes treated with insulin | 18 (17.6) | 4(8.0) | 0.2 |
| Post stress LVEF normal | 142 (75.5) | 109 (85.8) | **0.037** |
| DSPECT normal | 122(61.3) | 76(58.5) | 0.65 |
| All cause death | 52 (26.1) | 16 (12.3) | **0.004** |
| Cardiovascular death | 13 (6.7) | 1 (0.8) | **0.025** |
| 1st Cardiovascular event | 50 (25.8) | 22 (16.7) | 0.076 |

Table S3: Clinical descriptors by DSPECT Categories

| DSPECT  Variable n (%) | Overall  N=386 | Normal n=227 | Fixed only n=83 | Reversible n=29 | Mixed n=47 | p |
| --- | --- | --- | --- | --- | --- | --- |
| Age (years) | 73.4±9.6 | 73.9 ±9.9 | 72.7 ±9.6 | 74.5 ±9.1 | 72.3 ±8.1 | 0.57 |
| Females | 176 (45.4) | 129 (56.8) | 19 (23.2) | 18 (62.1) | 8 (17.0) | <0.001 |
| Diabetes treated with insulin | 29 (7.6) | 13 (5.8) | 9 (11.2) | 2 (6.9) | 5 (10.6) | 0.002 |
| hypertension | 294 (77.6) | 173 (77.9) | 57 (72.2) | 24 (82.8) | 37 (80.4) | 0.57 |
| End stage renal disease | 12(3.1) | 9 (4.0) | 3 (3.7) | 0 (0.0) | 0 (0.0) | 0.38 |
| Known coronary artery disease | 200 (51.5) | 76 (33.5) | 63 (76.8) | 20 (69.0) | 40 (85.1) | <0.001 |
| BHRR | 245 (63.1) | 140 (61.7) | 56 (68.3) | 15 (51.7) | 31 (66.0) | 0.4 |
| normal ps. LVEF | 281 (77.4) | 212 (97.2) | 35 (50.9) | 17 (63) | 17 (37) | < 0.001 |
|  | Outcomes | | | | | |
| All cause death | 90 (23.2) | 45 (19.8) | 28 (34.1) | 4 (13.8) | 13 (27.7) | 0.03 |
| Cardiovascular death | 20 (5.3) | 7 (3.2) | 9 (11.4) | 0 (0.0) | 4 (8.5) | 0.04 |
| 1^st^ Cardiovascular event | 85 (21.9) | 30 (13.5) | 22(27.8) | 12(41.4) | 20(42.6) | <0.001 |

Table S4: Reduced cox regression for cardiovascular death (adjusted for age and sex only)

|  | **HR** | **95% CI** | **p value** |
| --- | --- | --- | --- |
| **BHRR** | **9.52** | 1.27–71.33 | 0.028 |
| **Age (per 1 year)** | 1.01 | 0.97–1.06 | 0.581 |
| **Male sex** | 1.4 | 0.56–3.52 | 0.474 |
